# Molecular risk factors for locoregional recurrence in resected non‐small cell lung cancer

**DOI:** 10.1002/cam4.6165

**Published:** 2023-05-29

**Authors:** Wei Guo, Tao Zhang, Runze Li, Xiaoxi Chen, Jiaohui Pang, Hua Bao, Xue Wu, Yang Shao, Bin Qiu, Shugeng Gao, Jie He

**Affiliations:** ^1^ Department of Thoracic Surgery, National Cancer Center/National Clinical Research Center for Cancer/Cancer Hospital Chinese Academy of Medical Sciences and Peking Union Medical College Beijing China; ^2^ Department of Radiation Oncology, National Cancer Center/National Clinical Research Center for Cancer/Cancer Hospital Chinese Academy of Medical Science and Peking Union Medical College Beijing China; ^3^ Geneseeq Research Institute, Nanjing Geneseeq Technology Inc. Nanjing China; ^4^ School of Public Health Nanjing Medical University Nanjing China

**Keywords:** adjuvant therapy, biomarkers, distant recurrence, locoregional recurrence, NSCLC

## Abstract

**Background:**

Locoregional recurrence is of high risk and is associated with a poor prognosis in terms of OS for non‐small cell lung cancer (NSCLC). Local control is essential for radical cure of NSCLC. Previous studies have investigated the clinicopathological risk factors for locoregional recurrence, but the genomic biomarkers associated with locoregional recurrence have been inadequately studied.

**Methods:**

A total of 118 patients who underwent tumor resection with mutation‐detected tumor specimens were included. Tumor samples at surgery and pretreatment/postoperative blood samples were collected for mutational profiling.

**Results:**

Among 48 patients with disease recurrence, 46% developed locoregional recurrence (LR) and 75% developed distant metastasis (DM). The 3‐year actuarial risk of LR and DM was 25% and 43%, respectively. The first sites of failure were locoregional only (29%), locoregional and distant (10%), and distant only (61%). Patients with LR showed significantly higher ctDNA level than those with only DM at the time of initial recurrence. On multivariate analysis of baseline risk factors, the presence of allele frequency heterogeneity and baseline ctDNA shedding were found to be independently associated with a higher risk of LR. Patients with disruptive TP53 mutations had significantly lower LR‐free survival as compared to patients with wild‐type TP53 or nondisruptive mutations. EGFR mutations showed a favorable prognostic value for LR and is not induced by EGFR tyrosine kinase inhibitor therapy. Both disruptive TP53 mutation and EGFR mutation remained the significant prognostic factor after adjustment for histological type, pathologic nodal stage and adjuvant therapy.

**Conclusions:**

Nearly half of disease recurrences after surgery for NSCC involved locoregional sites. We identified genomic biomarkers from baseline tumor and ctDNA samples which showed promising prognostic value for LR only. This can help identify patients who had a higher risk of locoregional recurrence regardless of the risk of distant metastasis.

## Graphical abstract

Local control is essential for radical cure of NSCLC. Previous studies have investigated the clinicopathological risk factors for locoregional recurrence (LR), but the genomic biomarkers associated with LR have been inadequately studied. This study investigates the molecular biomarkers associated with LR regardless of distant metastasis in NSCLC patients, which could help improve local control and potentially improve overall survival for NSCLC patients.

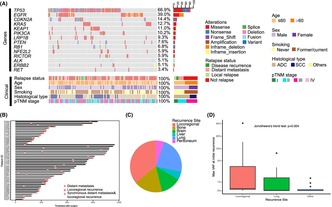

## 1 INTRODUCTION

Lung cancer is the primary cause of cancer‐related deaths worldwide, including in China. Non‐small cell lung cancer (NSCLC) accounts for ~85% of lung cancer cases,^1^ and curative radical resection is considered eligible for patients with Stages I, II, and certain Stage III NSCLCs.^2^ However, postoperative recurrence remains a significant challenge for many patients, including distant metastasis (DM), locoregional recurrence (LR), or both.^3^

Although DM is the most frequent first site of relapse in NSCLC patients who have undergone surgery, studies suggest that rates of local failure may be higher than previously thought, as many clinical trials only report on the first sites of failure. As such, rates of LR remain high.^4^, ^5^ For patients with LR after radical surgery without hematogenous spreading, sufficient local treatment can lead to a cure.^6^ However, most patients with locoregionally recurrent disease are not candidates for secondary radical surgery, underscoring the importance of effective local control for curative purposes. Several trials demonstrated that modern postoperative radiation therapy (PORT) reduced the risk of local recurrence with low toxic effects although it does not improve disease‐free or overall survival for pIIIA‐N2 NSCLC.^7^, ^8^ These findings highlight the need for comprehensive treatment approaches that address both local and distant disease control in NSCLC patients.

Most of the previous clinical trials also only concentrated on chemotherapy and/or targeted therapies as means of improving outcomes, with an emphasis on distant‐relapse‐free and overall survival.^9^, ^10^ In our previous publication, we focused on the utility of post‐treatment circulating tumor DNA (ctDNA) in recurrence monitoring, which is also regardless of recurrence type.^11^ Therefore, better understanding of patterns and risk factors of LR independently from DM may provide insight for selecting patients eligible for adjuvant radiation therapy. Several studies have investigated the clinicopathological risk factors for LR, but the molecular risk factors associated with LR have been inadequately studied. In this study, we investigated on pattern of recurrence and prognostic significance of genomic variants in patients with NSCLC who underwent complete resection.

## 2 MATERIALS AND METHODS

### 2.1 Patients and sample collection

Patients with NSCLC who underwent complete resection were enrolled at the Cancer Hospital of Chinese Academy of Medical Sciences from December 2018 to June 2021 and only patients with mutation‐detected tumor specimens were included (Figure S1). Four patients were excluded due to loss of follow‐up and one patient who was re‐confirmed as small cell lung cancer was also excluded. Four patients without available tumor samples were not included in this study. Furthermore, 21 patients with tumor samples but no somatic mutation detection in tumor samples or who did not pass sample quality control were excluded. In summary, a total of 118 patients with mutation‐detected tumor samples were included in this study and were approved by the Ethics Committee of Cancer Hospital, Chinese Academy of Medical Sciences, and Peking Union Medical College (NCC1843). All patients provided oral and written informed consent to participate and publication.

Presurgical mutational profiling was conducted using tumor tissue collected during surgery and pre‐treatment peripheral blood samples. Normal control for each patient was obtained from white blood cells collected from the buffy coat after plasma preparation. Follow‐up involved scheduling patients for computed tomography (CT) scans and blood collections every 3 months until CT scan results determined recurrences. LR was defined as disease recurrence at the surgical margin, ipsilateral hemithorax, or regional lymph nodes, whereas metastasis outside of the hemithorax or mediastinum, or to the contralateral lung was considered DM. Genetic testing was carried out by a centralized clinical testing center (Nanjing Geneseeq Technology Inc.), certified to meet CAP, CLIA, and ISO15189 standards.

### 2.2 Next‐generation sequencing and data processing

As described previously,^11^ targeted next‐generation sequencing (NGS) was performed on tumor tissue and plasma ctDNA specimens using a customized panel covering 139 lung cancer‐related genes (PULMOCAN™; Nanjing Geneseeq Technology Inc.). The KAPA Hyper Prep kit (Roche) was used for sequencing library preparation, following an optimized manufacturer's protocol. Libraries with unique indices were pooled in appropriate ratios for up to 2 μg of total input and quantified by qPCR using the KAPA Library Quantification Kit (KAPA Biosystems). Library fragment size was determined using Bioanalyzer 2100 (Agilent Technologies), and target‐enriched libraries were sequenced on HiSeq4000 NGS platforms (Illumina) following the manufacturer's instructions.

Quality control for FASTQ files was conducted using Trimmomatic^12^ to remove leading/trailing low‐quality or N bases. Qualified reads were then mapped to reference human genome (hg19) using Burrows‐Wheeler Aligner.^13^ and PCR duplicates were removed by Picard (Broad Institute) after local realignment around known indels and base quality recalibration using Genome Analysis Toolkit (GATK 3.4.0). Single‐nucleotide variations and insertions/deletions were detected using VarScan2^13^ with default parameters for tissue specimens. Mutations observed in ≥20 cancer cases in the COSMIC database were defined as hotspots, using a minimum variant allele frequency (minVAF) of 1% or 2% and minimum variant supporting reads of five or six, for hotspot mutations or other mutations, respectively. The detection of tumor‐specific mutations in pretreatment ctDNA was used to define baseline ctDNA shedding.

### 2.3 Classification of mutations

TP53 mutations were classified into two categories based on the predicted amino acid alterations. Disruptive mutations are nonconservative mutations (i.e., frameshift, nonsense, or splice‐site mutations) or stop codons in any region, and non‐disruptive mutations are conservative mutations. EGFR activating mutations were defined as mutations on exons encoding the tyrosine‐kinase domain of EGFR (exons 18 through 21).^14^

### 2.4 Allele frequency heterogeneity (AFH)

AFH status was categorized into two groups (presence or absence) according to the ratio of minVAF to the maximum‐somatic‐variant‐allele‐frequency (maxVAF) of a sample. The presence of AFH was defined as minVAF/maxVAF <0.1.

### 2.5 Statistical analysis

Disease‐free survival (DFS) was measured from the date of surgery until disease recurrence (locoregional or distant) or death occurred. Locoregional recurrence‐free survival (LRFS) was defined as the time between surgery and LR or death, while distant metastasis‐free survival (DMFS) was defined as the duration between surgery and DM or death. All time‐to‐event data (DFS, LRFS, and DMFS) were censored at the last follow‐up if the corresponding event had not occurred. Statistical analysis was performed using R software, version 4.1.2. Cox proportional hazards regression analysis and Kaplan–Meier estimates were used to evaluate the association of clinical variables and risk factors. Univariate analysis was conducted initially, with multivariate analysis subsequently performed using statistically significant clinical variables identified from univariate analysis. Two‐sided testing was used for all *p*‐values, with differences considered significant at *p* < 0.05.

## 3 RESULTS

### 3.1 Patient characteristics and baseline mutational profile

Out of 118 NSCLC patients who received surgical resection, 75 (63.6%) were male. The median age at the time of surgery was 61.5 years (Table 1; Table S1). A histologic analysis showed 79 (66.9%) adenocarcinoma, 34 (28.8%) squamous cell carcinoma, and 5 (10.2%) other types (two adenosquamous carcinomas and three large cell carcinomas).

**TABLE 1:** The clinicopathological characteristics of all patients.

Characteristics	No. (%)
No. of patients	118
Median follow‐up days (95% CI)	820 (84–1292)
Median age, years (range)	61.5 (36–80)
Sex	
Male	75 (63.6%)
Female	43 (36.4%)
Smoking status	
Former/current	66 (55.9%)
Never	52 (44.1%)
Histological type	
Adenocarcinoma	79 (66.9%)
Squamous cell carcinoma	34 (28.8%)
Adenosquamous carcinoma	2 (1.7%)
Large cell neuroendocrine carcinoma	3 (2.5%)
Pathological stage	
I	19 (16.1%)
II	43 (36.4%)
III	54 (45.8%)
IV	2 (1.7%)
T stage	
T1	16 (13.6%)
T2	64 (54.2%)
T3	29 (24.6%)
T4	9 (7.6%)
N stage	
N0	49 (41.5%)
N1/2	69 (58.5%)
Adjuvant therapy	
Chemotherapy	66 (55.9%)
Chemotherapy + radiotherapy	9 (7.6%)
Chemotherapy + targeted therapy	7 (5.9%)
Targeted therapy	6 (5.1%)
No	30 (25.4%)
Recurrence (%)	
No relapse	70 (59.3%)
Locoregional relapse	22 (18.6%)
Distant metastasis	36 (30.5%)

**FIGURE 1:**
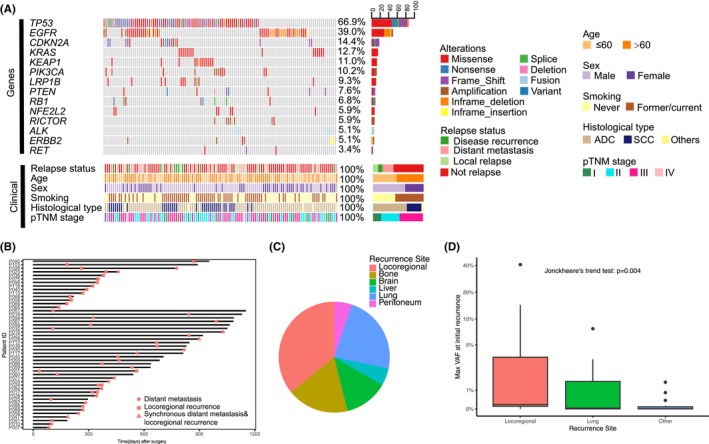
(A) The mutational profile of tumor samples from 118 non‐small cell lung cancer patients. (B) Swimming plot showing the time and duration of disease‐free survival. (C) Pie plot showing the sites of initial recurrence. Patients who developed their initial recurrence in the locoregional region, with or without simultaneous recurrence in other regions, were counted as “locoregional recurrence”. (D) Comparison of circulating tumor DNA level at the time of initial recurrence between different sites of recurrence.

In line with the previous study, TP53 (79/118, 66.9%) remained the most frequently mutated gene in this updated cohort (Figure 1A). EGFR (79/118, 39.0%) and CDKN2A (14.4%) were the next most frequently mutated genes. In consistence with our previous finding, pretreatment ctDNA shedding was significantly related to the pTMN stage and histological subtype.

### 3.2 Patterns of post‐surgical initial recurrence

Eighty‐eight patients (76%) received adjuvant therapy, including 66 patients with chemotherapy, 9 patients with chemoradiotherapy, 6 patients with targeted therapy, and 7 patients with chemotherapy plus targeted therapy. During a median follow‐up time of 820 days (range, 84–1292 days), disease recurrence (locoregional and/or distant disease recurrence) was observed in 48 patients (41%; Figure 1B). The median time from curative surgery to initial recurrence was 1029 (95% CI, 868–not reached) days. Twenty‐nine patients developed DM at their initial recurrence whereas 14 patients (29%) developed LR first. Five patients developed synchronous DM and LR. The most common sites of disease at initial recurrence were locoregional (*n* = 19, 16%), lung (*n* = 9, 8%), and bone (*n* = 7, 6%) metastases (Figure 1C). Locoregional disease recurrence occurred in 22 patients, with a median time from surgery to recurrence of 334 days (range, 138–1029 days) for those patients affected. The 3‐year actuarial locoregional disease recurrence risk was 25% (95% CI, 14–36%; see Figure S2A). Distant recurrence was detected in 36 patients, with a median time from surgery to recurrence of 361 days (range, 33–1029 days) for those patients affected. The 3‐year actuarial distant failure risk was 43% (95% CI, 28%–55%; see Figure S2B).

Among 48 recurrence patients, 41 had ctDNA available at initial recurrence. Patients who experienced their first recurrence in the local region, with or without simultaneous recurrence in other regions, were classified as having “locoregional recurrence”. Patients with LR showed significantly higher ctDNA levels than those with metastasis only (Figure S3). Of note, we observed a substantial decrease in ctDNA level at the time of recurrence from LR to lung metastasis and other DM (Figure 1D), suggesting that recurrence site location may influence the liquid diagnostic accuracy in NSCLC.

### 3.3 Baseline risk factors with increased risk of LR

Our previous results showed that postsurgical ctDNA, post‐adjuvant therapy ctDNA, or longitudinal serial ctDNA serve as prognostic markers in DFS of resected NSCLC.^11^ However, these factors strata both have LRFS (Figure S4A) and DMFS (Figure S4B). In this study, we sought to identify baseline risk factors that are prognostic to only LR.

**FIGURE 2:**
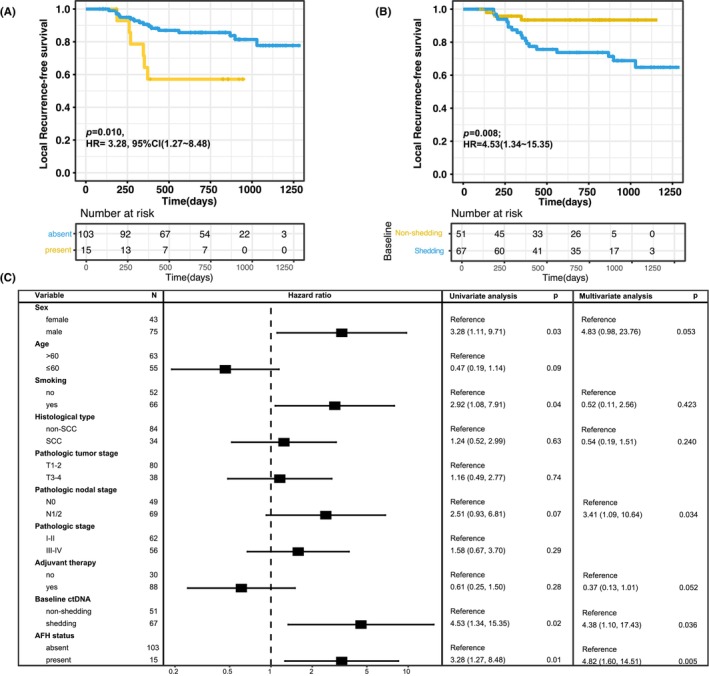
(A) Kaplan–Meier curve of locoregional recurrence‐free survival (LRFS) in patients stratified by allele frequency heterogeneity status. (B) Kaplan–Meier curve of LRFS in patients stratified by baseline circulating tumor DNA detection. (C) Univariate and multivariate cox regression analysis of baseline risk factors LRFS.

According to time‐dependent analysis, the risk of LR was higher for patients with the presence of AFH (hazard ratio [HR]: 3.28; 95% CI: 1.27–8.48; *p* = 0.01; Figure 2A) and pretreatment ctDNA shedding (HR: 4.53; 95% CI: 1.34–15.35; *p* = 0.02; Figure 2B). Other variables associated with a higher rate of LR on univariate analysis were male gender (*p* = 0.02) and smoking (*p* = 0.04; Figure 2C). A multivariate Cox proportional hazard analysis suggested that the presence of AFH and baseline ctDNA shedding were independently associated with LR after adjustment for Sex, smoking status, histological type, pathologic nodal stage, and adjuvant therapy. We found no difference in LRFS (*p* = 0.3; Figure S6A) or types of LR (*p* = 0.36; Fisher's exact test) between patients undergoing sublobar resection (wedge resection or segmentectomy) and those undergoing lobectomy or pneumonectomy. The proportion of LR did not exhibit a preference concerning the pathological stage (*p* = 0.59; Fisher's exact test; Figure S6B).

The risk factors for distant failure were higher pathological stage (Stages III–IV) and N stage (N1‐2) disease (Figure S5B), while the risk of distant failure was similar for patients with different baseline ctDNA shedding status and tumor AFH status.

### 3.4 Only disruptive mutations on TP53 gene were risk factors for LR

**FIGURE 3:**
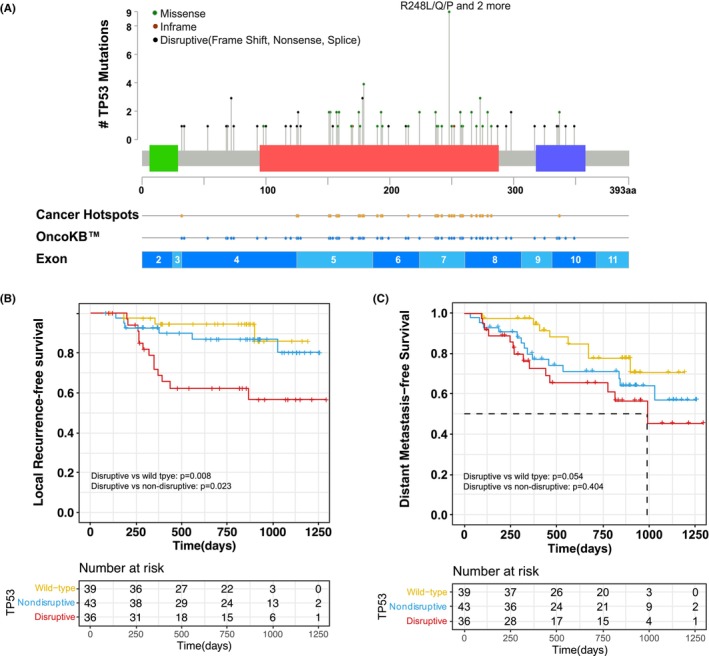
(A) Lollipop figure of TP53 mutations detected in baseline tumor samples of all patients. Green dots indicate missense mutations, red dots indicate inframe indel mutations and black dots indicate disruptive (i.e., frame Shift, nonsense, splice‐site) mutations. All disruptive TP53 mutations in this cohort were included in Cancer Hotspots or OncoKB databases. (B) Kaplan–Meier curve of locoregional recurrence‐free survival (LRFS)in patients stratified by TP53 mutation categories. (C) Kaplan–Meier curve of distant metastasis‐free survival (DMFS) in patients stratified by TP53 mutation categories.

In this cohort, patients with mutant TP53 had significantly worse DFS (HR: 2.17; 95% CI: 1.08–4.36; *p* = 0.03; Figure S7A) than those with wild‐type TP53 and had a trend of worse LRFS (HR: 3.31; 95% CI: 0.98–11.21; *p* = 0.042; Figure S7B) and DMFS (HR: 1.98; 95% CI: 0.90–4.37; *p* = 0.088; Figure S7C). Since different types of mutations in the TP53 gene are known to have different effects on the functionality of the protein, we further classified TP53 mutation into non‐disruptive and disruptive based on their impact on protein structure (Figure 3A). All disruptive TP53 mutations in this cohort were included in Cancer Hotspots or OncoKB databases.

As compared to patients with wild‐type TP53, the 36 patients with disruptive mutations had significantly lower LRFS (HR: 5.45; 95% CI: 1.55–19.17; *p* = 0.008), but the 43 patients with nondisruptive mutations did not (HR: 1.78; 95% CI: 0.44–7.12; *p* = 0.42) (Figure 3B). The group with disruptive TP53 mutations also had shorter LRFS than did the group with non‐disruptive TP53 mutations (HR: 3.07; 95% CI: 1.16–8.11; *p* = 0.023). In multivariate analyses involving Cox proportional‐hazards models, as compared with the absence of a TP53 mutation, the presence of TP53 disruptive mutation (HR: 7.84; 95% CI: 2.00–30.79; *p* = 0.003; Figure S7D) remained significantly associated with decreased survival after adjustment for smoking status, histological type, pathologic nodal stage, and adjuvant therapy. Nevertheless, patients with disruptive or non‐disruptive TP53 mutation did not show significant differences in DMFS than those with wildtype TP53 (Figure 3C), which emphasized the prognostic value of TP53 disruptive mutations on LR.

### 3.5 EGFR mutation as a favorable prognostic factor for LR

**FIGURE 4:**
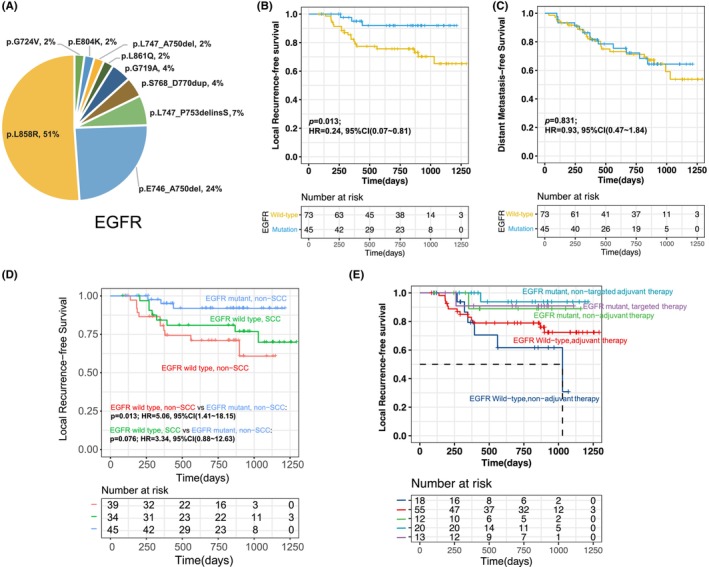
(A) EGFR mutations identified in baseline tumor samples of all patients. (B) Kaplan–Meier curve of locoregional recurrence‐free survival (LRFS) in patients stratified by EGFR status. (C) Kaplan–Meier curve of distant metastasis‐free survival (DMFS)in patients stratified by EGFR status. (D) Kaplan–Meier curve of LRFS in patients stratified by EGFR status and histological subtypes. (E) Kaplan–Meier curve of LRFS in patients stratified by EGFR status and adjuvant therapy status.

EGFR mutations were identified in 45 (38%) patients. Among those, most of (43/45, 96%) mutations were activating mutations, including L858R (*n* = 23, 51%), exon19 del (*n* = 15, 33%), exon 20 ins (*n* = 2, 4%) and G719S (*n* = 2, 4%) and L861Q (*n* = 1, 2%) (Figure 4A). The presence of activating EGFR mutation was significantly associated with increased LRFS (HR: 0.24; 95% CI: 0.07–0.81; *p* = 0.013; Figure 4B), whereas DMFS showed no difference (*p* = 0.831; Figure 4C) in EGFR‐positive patients and wildtype EGFR. We also compared LRFS between non‐SCC patients with EGFR‐positive or wildtype EGFR as all SCC patients had wildtype EGFR. The EGFR‐positive non‐SCC patients showed a significant better LRFS than wildtype EGFR non‐SCC patients (HR: 5.06; 95% CI: 1.41–18.15; *p* = 0.013) and a trend of better LRFS than SCC patients (HR: 3.34; 95% CI: 0.88–12.63; *p* = 0.076; Figure 4D). Furthermore, this favorable prognostic value of EFGR mutation on LR is not induced by EGFR‐TKI therapy as no significant difference in LRFS was identified among EFGR mutation patients treated with adjuvant EGFR TKI therapy, adjuvant chemo‐/radio‐ therapy, or no adjuvant therapy. EGFR mutation remained the significantly better prognostic factor after adjustment for histological type, pathologic nodal stage, and adjuvant therapy (HR: 0.19; 95% CI: 0.04–0.86; Figure S8).

## 4 DISCUSSION

In the antecedent cohort study, we examined NSCLC patients treated with surgical resection with or without adjuvant therapy to evaluate the utility of ctDNA in disease monitoring and treatment determination.^11^ In the current study, our updated cohort of 118 patients was utilized to further investigate recurrence patterns and explore risk and prognostic factors associated with LR in resected NSCLC patients. Among the 48 patients who experienced disease recurrence, 46% developed LR and exhibited significantly elevated levels of ctDNA during recurrence. Two baseline genomic factors, baseline ctDNA shedding, and baseline AFH, were both independently related to a higher risk of LR. Furthermore, non‐disruptive‐mutated/wild‐type TP53 or mutated EGFR were identified as favorable prognostic biomarkers for LR.

The cohort utilized in this study was an updated version of the cohort from our previous publication. Although the majority of enrolled patients were the same between the two studies, two distinct patient enrollment criteria were employed. Patients who had insufficient post‐treatment plasma collection and were excluded from the previous publication were re‐enlisted in this study. Furthermore, the endpoint of the previous study was DFS, which was defined as the time between surgery and the diagnosis of relapse or death. This led to the exclusion of patients who experienced initial recurrence from further analysis. However, in this study, patients were followed up until both LR and DM or death occurred.

The risk of LR after surgery for NSCLC patients is generally considered to be lower than the risk of distant recurrence. However, the reported rate of local failure widely varies across studies, for example from 8% to 24% in studies with a relatively large number of patients.^15^, ^16^ The risk of LR may be underestimated and overshadowed by the risk of distant recurrence since most studies only report the initial sites of failure, and distant recurrence frequently occurs first after surgery.^4^ In this study, 71% of relapsed patients had a distant recurrence, but 46% had a LR and the actuarial risk of locoregional disease recurrence was 25% at 3 years, which appears sufficiently high to warrant further examination into adjuvant local therapy. Furthermore, studies have shown that overall survival is comparable in patients who only experience local recurrence to those who develop distant recurrence.^17^ Although the effectiveness of local treatments in improving survival may be limited by the risk of distant failure, recent studies suggest that modern PORT can significantly improve survival as an adjunct to postoperative chemotherapy for specific patients, such as those with N2 nodal disease, after tumor resection.^18^, ^19^ Therefore, if high‐risk patients for LR could be accurately identified, it would further enhance the therapeutic outcome and potentially facilitate clinical trials exploring the role of postoperative radiotherapy in resected NSCLC.

Several clinical and pathologic factors have been linked to a higher risk of LR in complete resected NSCLC, including pathological tumor size, lymphovascular invasion, and nodal disease.^16^, ^17^, ^20^ Several studies including our previous publication have also shown that several genomic biomarkers, such as gene expression profiles, DNA methylation markers, postoperative ctDNA, and genetic mutations can distinguish patients at particularly high risk of disease recurrence.^11^, ^21^, ^22^, ^23^, ^24^ These biomarkers hold the potential to guide adjuvant chemotherapy after surgery. However, they have not yet differentiated between locoregional and distant disease recurrence or shown the potential to guide the use of postoperative radiotherapy. In this study, we found the presence of AFH, baseline ctDNA shedding, disruptive TP53 mutation, and wildtype EGFR to be independently associated with only a shorter LRFS, but not DMFS. These biomarkers may serve as prognostic factors for only locoregional failure in resected NSCLC, which could aid in identifying patients who may benefit from adjuvant radiotherapy or chemoradiotherapy.

TP53 is the most extensively investigated prognostic marker in NSCLC and was shown to be a prognostic factor in localized‐ and advanced‐stage NSCLC.^11^, ^25^ We found that although TP53 mutations were related to a significantly higher risk of disease recurrence, only disruptive TP53 mutations were significantly associated with shorter LRFS and patients with TP53 non‐disruptive mutations had similar LRFS as those with wildtype TP53. Nevertheless, TP53 non‐disruptive and disruptive mutations showed relatively worse DMFS. This finding supports the existing evidence that different types of TP53 mutations behave differently and affect pathways involved in maintaining genomic integrity that involve p53. The biological effects of TP53 mutations may also be influenced by the presence or absence of the remaining wild‐type allele and by the gain of function of some mutations.^26^, ^27^, ^28^ Further studies are needed to explore how different types of TP53 mutations interact with other molecular markers and factors to impact the risk of LR.

EGFR was one of the most commonly mutated genes in East Asian‐ancestry NSCLC.^29^, ^30^ The prognostic significance of EGFR alteration in NSCLC is still a matter of debate. A meta‐analysis study demonstrated that EGFR alterations were not associated with prognosis in patients who underwent NSCLC resection. However, several studies have revealed that resected EGFR‐positive NSCLC had a comparable recurrence risk or a higher risk of metastatic recurrence compared to wild‐type EGFR NSCLC.^31^, ^32^, ^33^ Furthermore, Saw et al. showed that patients with EGFR‐positive NSCLC who underwent curative surgical procedures had significantly better overall survival.^31^ Our data demonstrated that patients with EGFR mutations were significantly associated with better LRFS but had similar DFS or DMFS than those with wildtype EGFR. This difference in LRFS was not induced by the use of EGFR TKI adjuvant treatment as EGFR‐positive patients with no adjuvant therapy or non‐TKI adjuvant therapy showed similar favorable LRFS. Therefore, EGFR mutations may serve as a favorable prognostic factor for LR, but no DMFS.

Our study has several limitations. This is a single‐site retrospective study including only Chinese patients, which may limit the generalizability of the data. The sample size was relatively small considering the complexity in the pathological stage and adjuvant treatment of the cohort and the results should be confirmed in larger prospective studies. Furthermore, although the follow‐up period is relatively short, our study had already captured a significant number of recurrence events, with 40.7% of patients experiencing recurrence. We believe that longer follow‐up periods may not necessarily lead to significantly different results.

In conclusion, we identified genomic biomarkers from baseline tumor and ctDNA samples which showed promising prognostic value for LR only. This may help identify patients who had a higher risk of LR regardless of the risk of DM. Prospective studies are needed to validate our risk and prognostic factors with the aim of identifying patients who will benefit from adjuvant radiotherapy.

## AUTHOR CONTRIBUTIONS

**Wei Guo:** Conceptualization (equal); formal analysis (equal); investigation (equal); methodology (equal); visualization (equal); writing – original draft (equal). **Tao Zhang:** Conceptualization (equal); formal analysis (equal); methodology (equal); writing – original draft (equal). **Runze Li:** Data curation (equal); formal analysis (equal); investigation (equal); resources (equal). **Xiaoxi Chen:** Formal analysis (equal); software (equal); visualization (equal); writing – original draft (equal). **Jiaohui Pang:** Formal analysis (equal); visualization (equal). **Hua Bao:** Project administration (equal); supervision (equal); writing – review and editing (equal). **Xue Wu:** Project administration (equal); supervision (equal); writing – review and editing (equal). **Yang Shao:** Project administration (equal); supervision (equal). **Bin Qiu:** Conceptualization (equal); formal analysis (equal); funding acquisition (equal); investigation (equal); methodology (equal); project administration (equal); resources (equal); writing – review and editing (equal). **Shugeng Gao:** Conceptualization (equal); funding acquisition (equal); project administration (equal); supervision (equal). **Jie He:** Funding acquisition (equal); project administration (equal); resources (equal); supervision (equal).

## FUNDING INFORMATION

This study was funded by grants from the Beijing Municipal Science & Technology Commission (No. Z191100006619116) and Beijing Hope Run Special Fund of Cancer Foundation of China (No. LC2020A05).

## CONFLICT OF INTEREST STATEMENT

Xiaoxi Chen, Jiaohui Pang, Hua Bao, Xue Wu, and Yang Shao are employees of Nanjing Geneseeq Technology Inc. All remaining authors have declared no conflicts of interest.

### ETHICS STATEMENT

The study was approved by the Ethics Committee of Cancer Hospital, Chinese Academy of Medical Sciences and Peking Union Medical College. All patients provided oral and written informed consent to participate and publication.

## Supporting information


Figures

Table S1.

## Data Availability

All the data in the paper or in the supplementary materials are free to obtain. Raw data that support the findings of this study are available from the corresponding author upon reasonable request.
