# Supplementary material for: Molecular risk factors for locoregional recurrence in resected non‐small cell lung cancer
Source: Cancer Med. 2023 May 29;12(14):15026–36. doi: 10.1002/cam4.6165 (PMC10417202; doi:10.1002/cam4.6165)
Supplement: Supplementary file 1 — Figures [file CAM4-12-15026-s002.pdf]

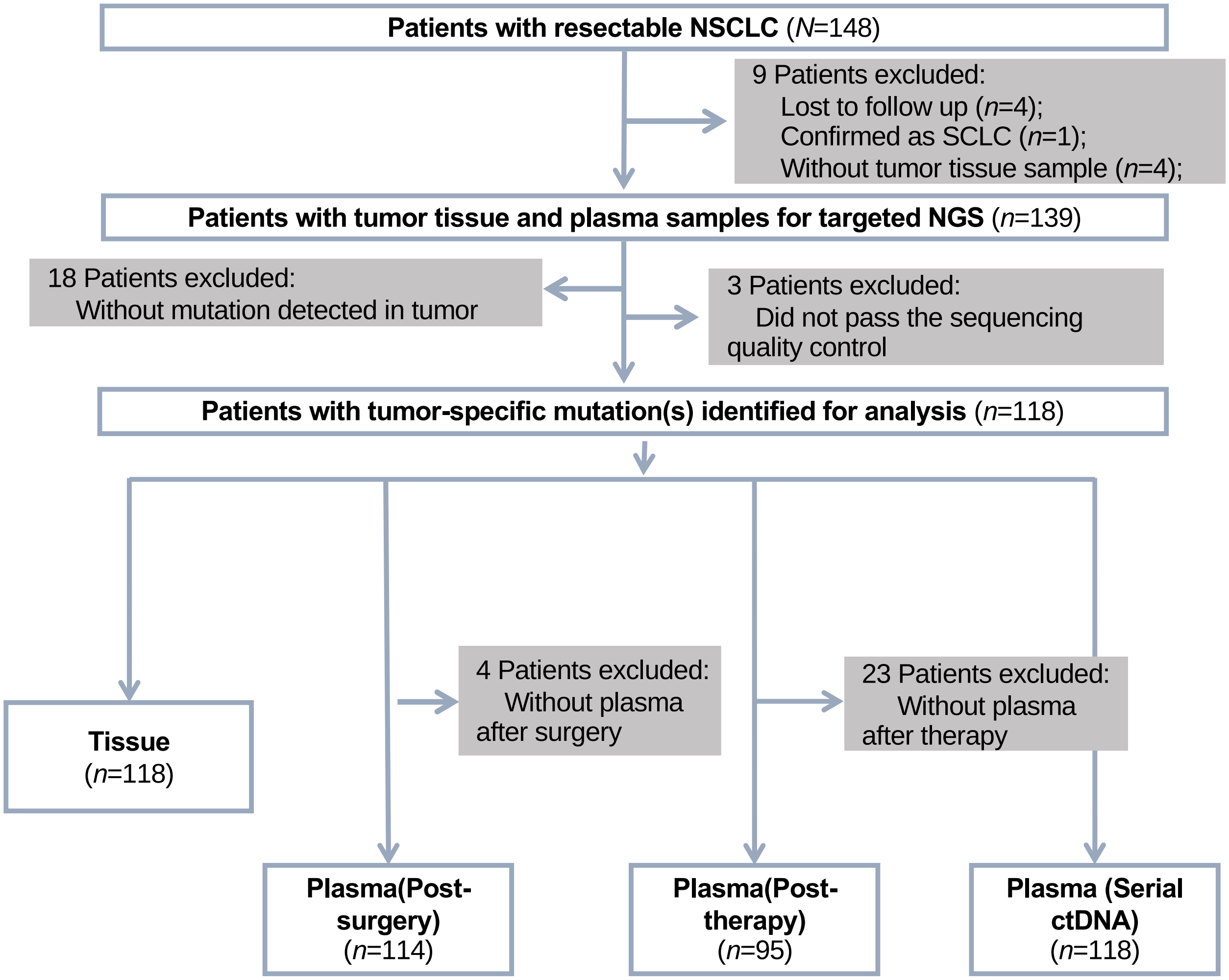

Fig S1. Patient enrollment and sample selection flowchart

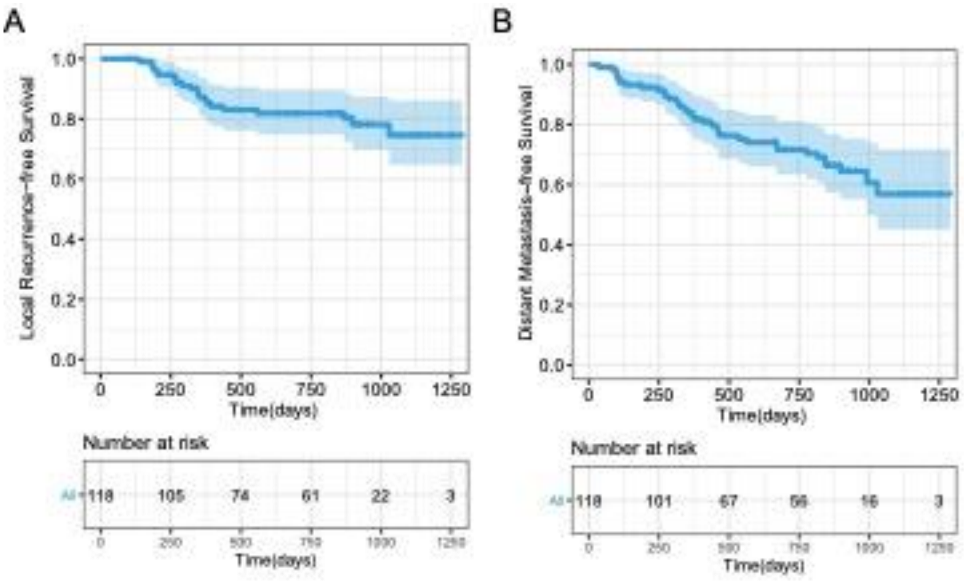

Fig S 2. Kaplan–Meier curve of locoregional recurrence-free survival (LRFS)(A) and distant metastasis-free survival (DMFS) (B) of all patients.

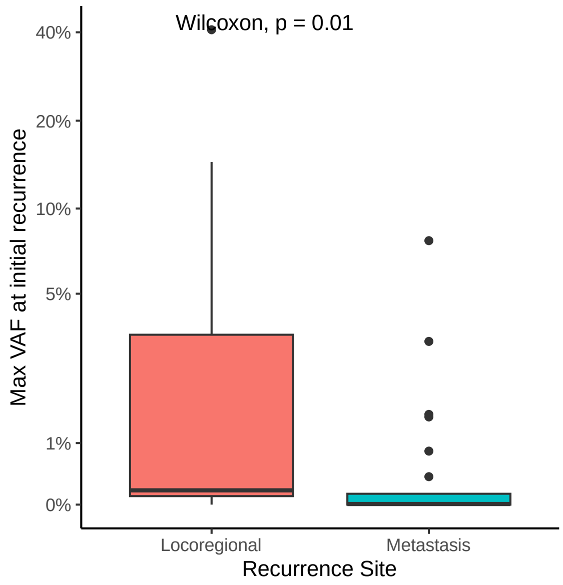

Fig S3. Comparison of ctDNA level at the time of initial recurrence between locoregional recurrence and metastasis only patients

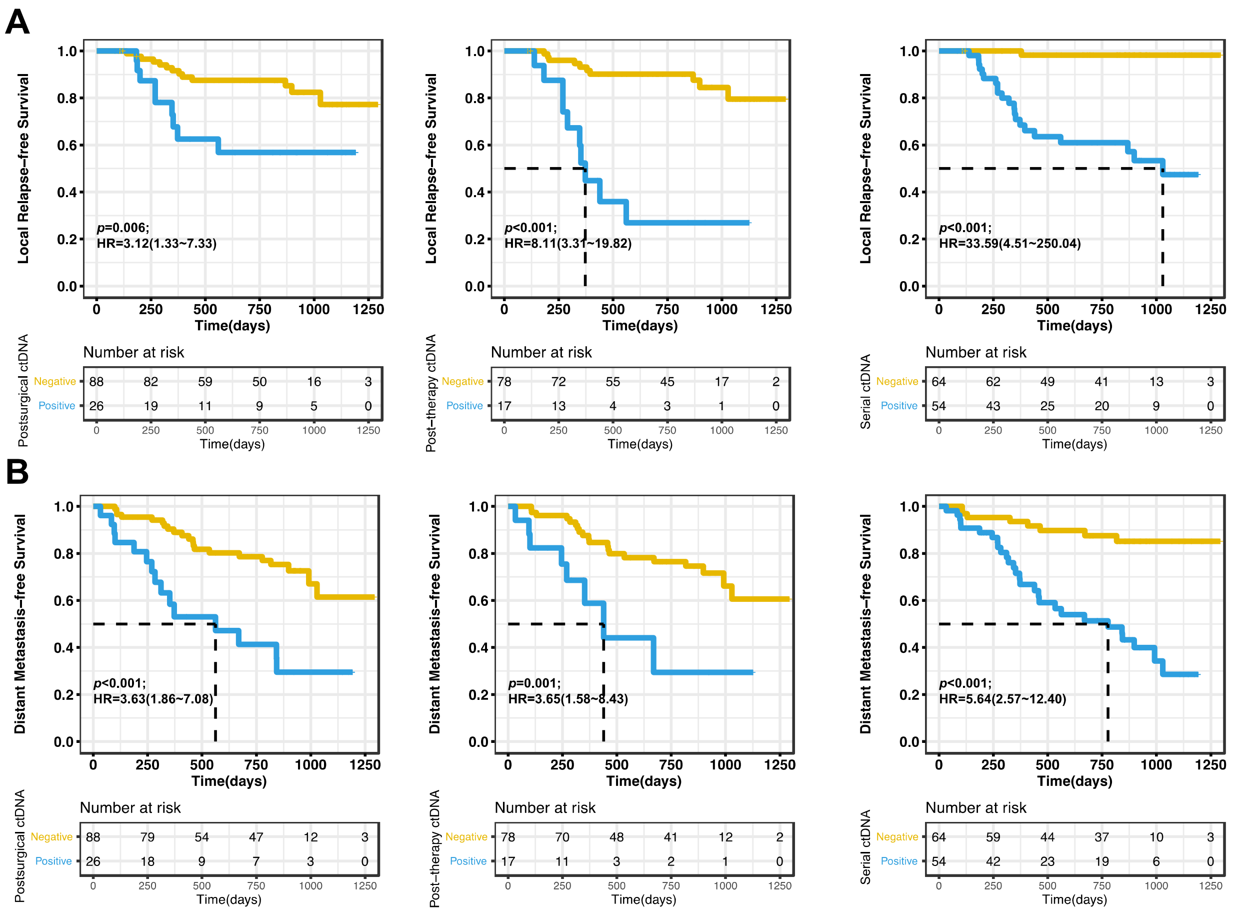

Fig S4. Kaplan–Meier curve of locoregional recurrence-free survival (LRFS)(A) and distant metastasis-free survival (DMFS) (B) in patients stratified by postsurgical ctDNA status, post-adjuvant therapy ctDNA status or longitudinal serial ctDNA status.

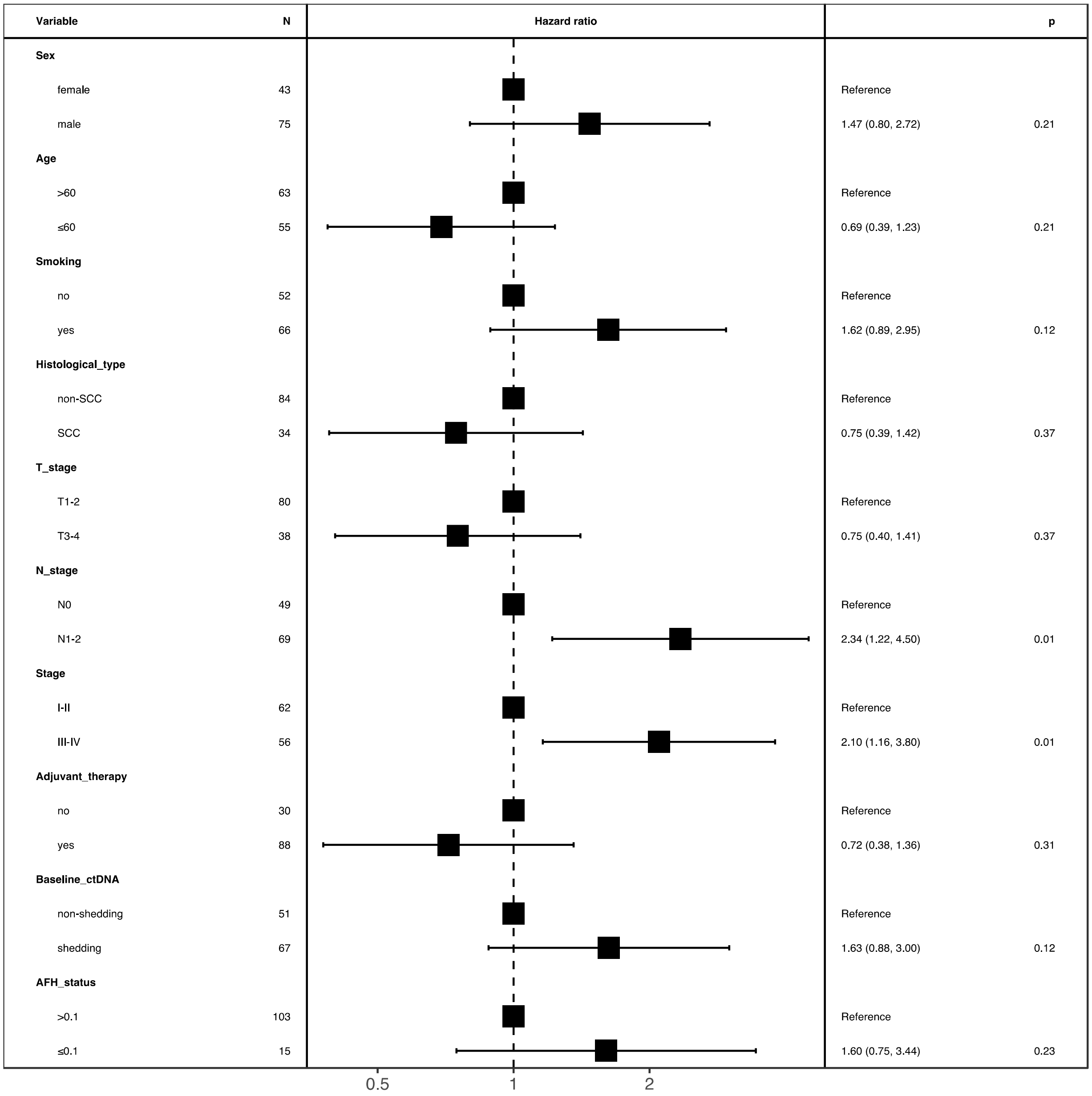

Fig S5A. Univariate cox regression analysis of baseline risk factor for disease-free survival (DFS)

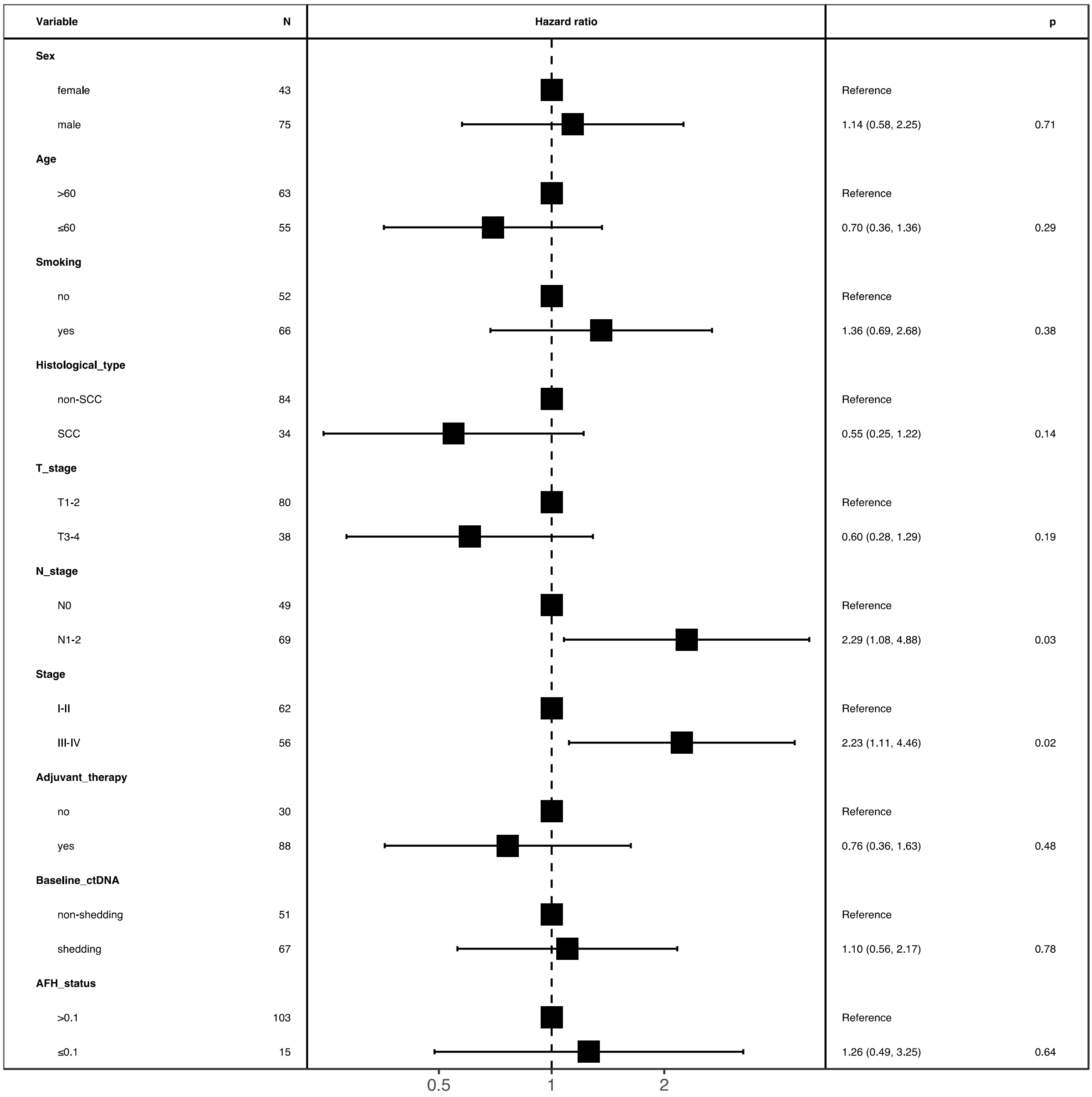

Variable	N		p
**Sex**			
female	43	Reference	
male	75	1.14 (0.58, 2.25)	0.71
**Age**			
>60	63	Reference	
≤60	55	0.70 (0.36, 1.36)	0.29
**Smoking**			
no	52	Reference	
yes	66	1.36 (0.69, 2.68)	0.38
**Histological_type**			
non-SCC	84	Reference	
SCC	34	0.55 (0.25, 1.22)	0.14
**T_stage**			
T1-2	80	Reference	
T3-4	38	0.60 (0.28, 1.29)	0.19
**N_stage**			
N0	49	Reference	
N1-2	69	2.29 (1.08, 4.88)	0.03
**Stage**			
I-II	62	Reference	
III-IV	56	2.23 (1.11, 4.46)	0.02
**Adjuvant_therapy**			
no	30	Reference	
yes	88	0.76 (0.36, 1.63)	0.48
**Baseline_ctDNA**			
non-shedding	51	Reference	
shedding	67	1.10 (0.56, 2.17)	0.78
**AFH_status**			
>0.1	103	Reference	
≤0.1	15	1.26 (0.49, 3.25)	0.64

Fig S5B. Univariate cox regression analysis of baseline risk factors for distant metastasis-free survival (DMFS)

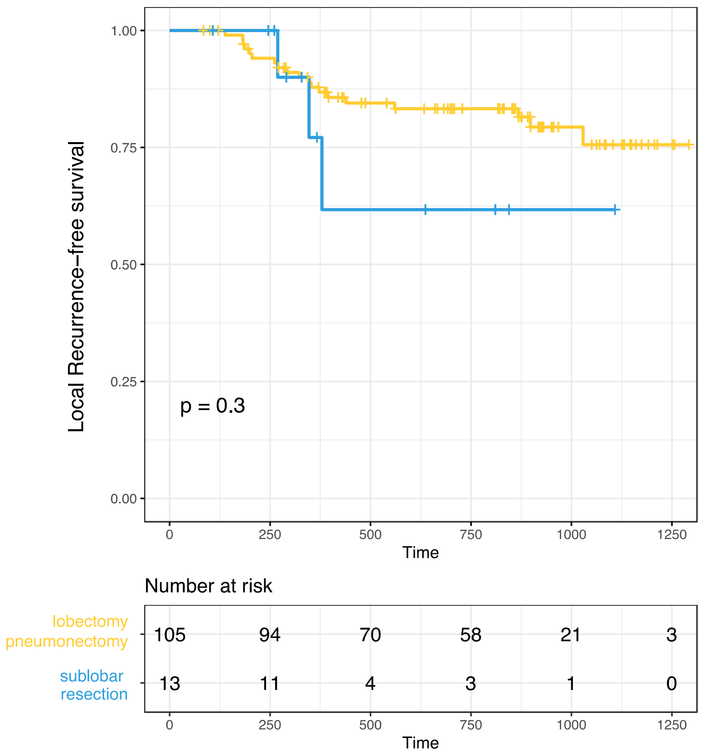

Fig S6A. Kaplan–Meier curve of LRFS in patients stratified by operative methods.

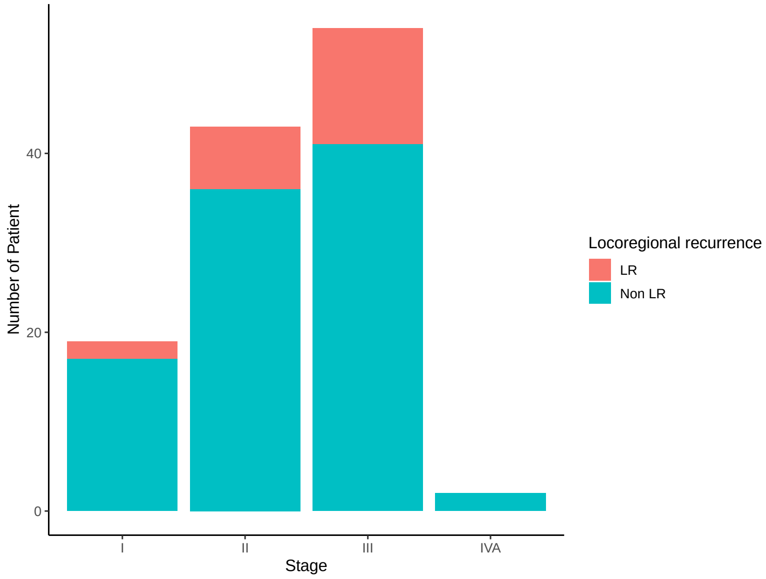

Fig S6B. Ratio of the locoregional recurrence in relation to the pathological stage.

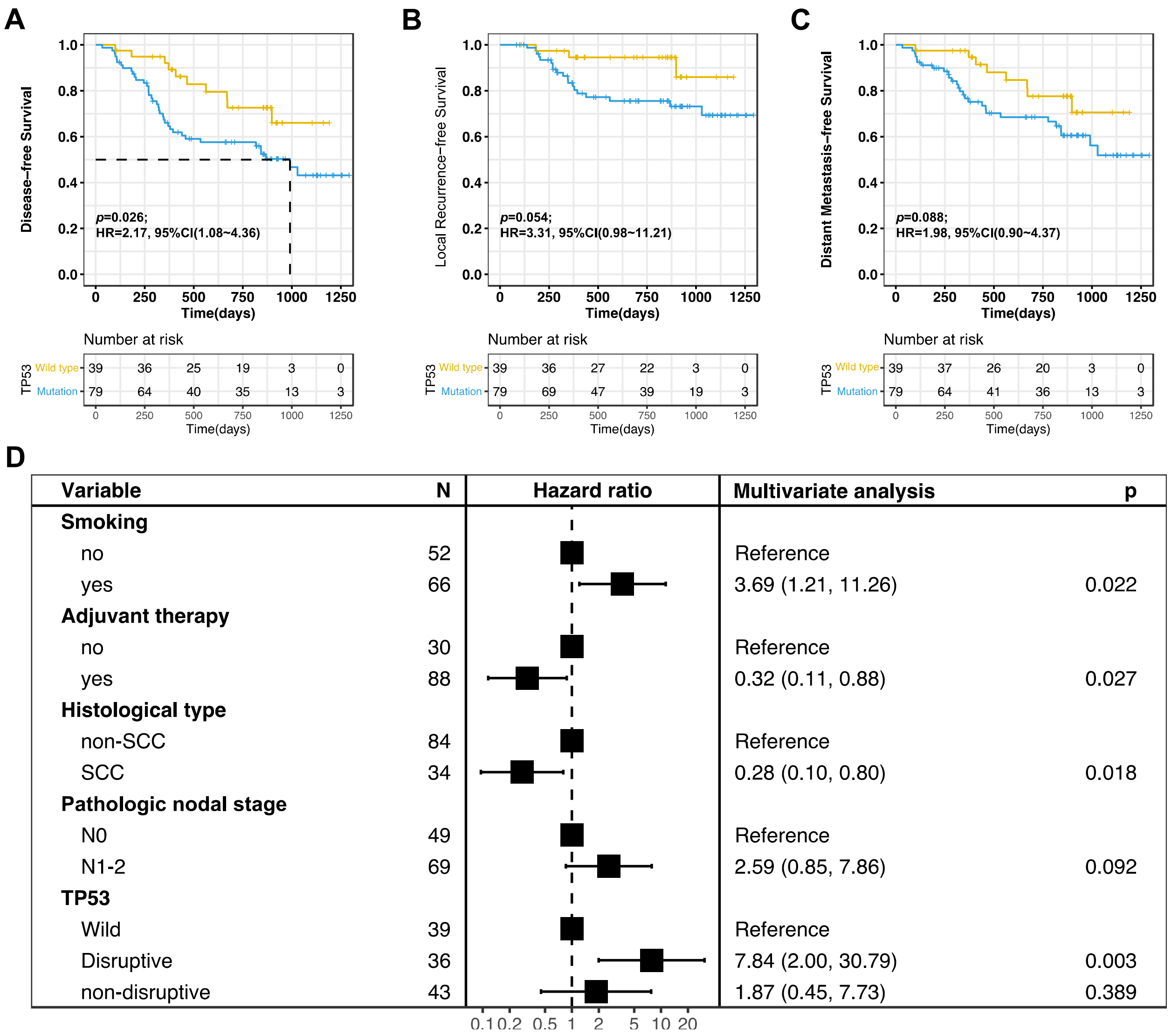

Fig S7. Kaplan–Meier curve of DFS (A), LRFS (B) and DMFS (C) in patients stratified by TP53 status. (D) Multivariate cox regression analysis of selected prognostic factors and TP53 mutation categories for LRFS

Variable	N	Hazard ratio	Multivariate analysis	p
**Adjuvant therapy**				
No	30		Reference	
chemo/radio	75		0.36 (0.13, 0.95)	0.04
Targeted	13		0.46 (0.04, 5.73)	0.54
**Histological type**				
non-SCC	84		Reference	
SCC	34		0.56 (0.22, 1.43)	0.23
**Pathologic nodal stage**				
N0	49		Reference	
N1-2	69		3.48 (1.18, 10.33)	0.02
**EGFR status**				
wild type	73		Reference	
mutant	45		0.19 (0.04, 0.86)	0.03

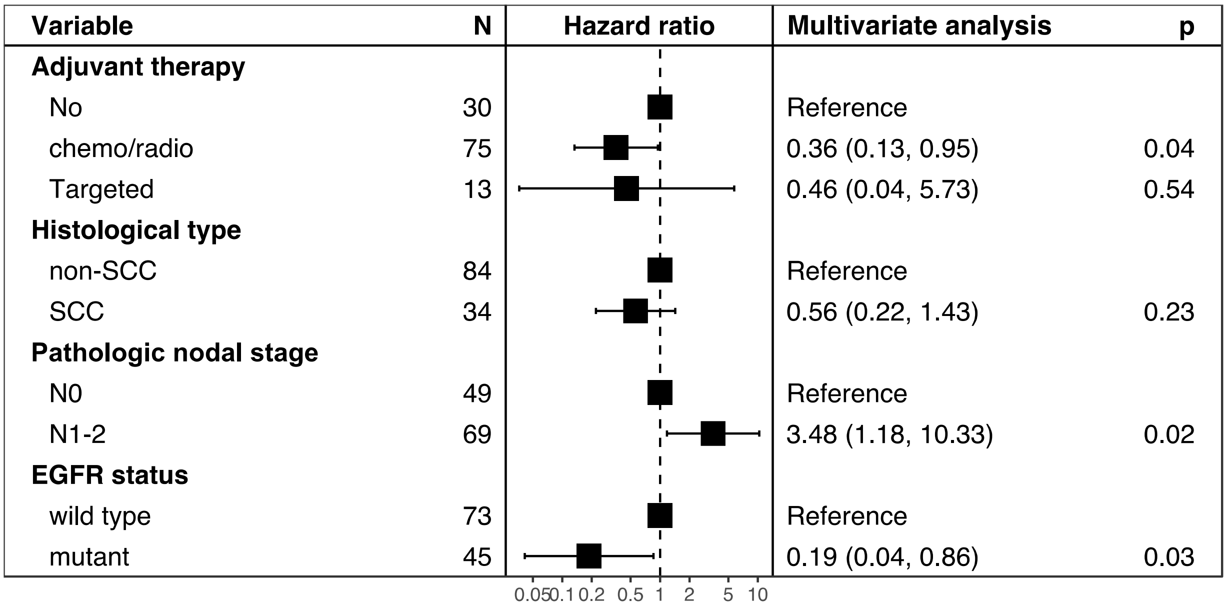

Fig S8. Multivariate cox regression analysis of selected prognostic factors and EGFR status for LRFS
